# Growth Characteristics, Growing Medium Properties, and Leaf Nutrient Contents of *Silene compacta* Fisch. Seedlings at Two Growing Periods Under Standardized Nursery Conditions

**DOI:** 10.3390/plants15142232

**Published:** 2026-07-22

**Authors:** Selma Kösa, Sibel Mansuroğlu

**Affiliations:** Department of Landscape Architecture, Faculty of Architecture, Akdeniz University, Antalya 07070, Turkey; smansur@akdeniz.edu.tr

**Keywords:** *Silene compacta* Fisch., container-grown seedlings, production period, leaf nutrient status, vegetative growth, ornamental nursery

## Abstract

Despite the increasing interest in *Silene compacta* Fisch. as a landscape plant, its greenhouse cultivation protocols remain poorly explored, leaving a critical gap in optimizing seedling production duration and efficiency. Therefore, this study aimed to compare the growth characteristics, growing medium properties, and leaf nutrient contents of *S. compacta* seedlings grown for two different periods (2.5 and 4.5 months) under standardized nursery conditions using a single pot type, a uniform growing medium, and a fixed fertilization program. Seedlings were grown simultaneously under greenhouse conditions, and final measurements were made at the end of each assigned growing period. Differences between the two growing periods were evaluated using independent samples *t*-tests based on replicate means. Several growth traits, including leaf number, leaf area, root number, stem diameter, and root and shoot biomass, were significantly higher at 4.5 months than at 2.5 months, whereas plant height, plant width, lateral branching traits, leaf dimensions, and root length did not differ significantly between the two periods. In the growing medium, pH was higher at 4.5 months, whereas total nitrogen and several micronutrients were higher at 2.5 months. Leaf N, P, K, Mn, and Cu concentrations were also higher at 2.5 months, whereas leaf Ca concentration was higher at 4.5 months. Overall, the results indicate that the 2.5-month growing period provided sufficient seedling development within a shorter production time and may offer significant economic and practical advantages in terms of compact appearance, lower input requirement, and earlier saleable seedling production under the nursery conditions tested.

## 1. Introduction

In Turkey, the genus *Silene* (family Caryophyllaceae) naturally occurs with a total of 147 taxa, including 129 species, 29 subspecies, and 9 varieties, of which 52 are endemic [1]. *Silene compacta* Fisch., a species native to Western, Southern, Central, Northern, Eastern, and Southeastern Anatolia and occurring at altitudes ranging from 0 to 2100 m, has been found suitable as an ornamental plant due to its characteristics, such as being a biennial or short-lived perennial, having erect stems that can reach up to 120 cm, and bearing numerous pink flowers on its inflorescences [2,3,4,5,6,7,8].

Despite its striking flowers and potential for use in various garden designs, studies determining the cultivation and market-ready period for *S. compacta* seedlings to be available from nurseries are quite limited. For effective nursery management and an economical production process, it is crucial to have basic knowledge regarding the species’ responses to growing media and fertilization programs, as well as its growth performance under specific ecological conditions [9,10,11].

Determining an appropriate growing period in nursery production is important for seedling morphology, visual quality, and production efficiency. Inadequate production duration may limit balanced plant development, whereas unnecessarily prolonged production may increase time and input use. Therefore, evaluating seedling growth together with leaf nutrient contents and growing medium properties at different production periods can contribute useful information for nursery management of *S. compacta*.

For herbaceous species with relatively short life cycles, such as *S. compacta*, container production conditions are important because irrigation, fertilization, and substrate characteristics can influence plant growth and nutrient dynamics during cultivation. Warsaw et al. [12] reported that plants grown in pots may lose part of the applied fertilizers during irrigation due to the small substrate volume and rapid drainage, and that fertilizer and water management can significantly affect plant growth and nutrient efficiency. Argo and Biernbaum [13] and Scagel [14] emphasized that a series of interacting factors influence the availability of nutrients from container medium for plant uptake throughout production. Some studies have also shown that the physical and chemical properties of growing media create different effects on growth and flowering depending on the plant species [15,16,17,18].

Leaf nutrient contents can also provide useful information for interpreting plant performance during nursery production. Some studies have emphasized that leaf nitrogen (N) and phosphorus (P) contents affect plant growth processes [19,20,21,22]. In this context, Liu et al. [21] highlighted that the application of nitrogen (N) and phosphorus (P) influences root traits (branching intensity and root tissue density) and leaf traits (net photosynthetic rate, stomatal conductance, and transpiration rate). Gupta and Dilta [23] reported that the growing medium and pot size can significantly affect plant growth and flowering traits. Kanwar et al. [24] emphasized that the composition of the growing medium and the applied fertilization program play an important role in determining plant growth, flowering, and pot presentation performance. Scagel [14] noted that differences in Mg and Fe concentrations in plants arise as a result of varying plant growth rates. Abravesh et al. [25] on the other hand, reported that foliar selenium applications positively affect anatomical traits such as stem and vascular cylinder diameter, leading to enhanced vegetative growth and higher photosynthetic efficiency.

A review of the available literature indicates that nursery-based information on *S. compacta*, particularly regarding seedling growth at different production periods under standardized conditions, remains limited. In this context, comparing different growing periods under a uniform pot size, growing medium, and fertilization program can help clarify the production response of this species in nursery conditions.

Accordingly, the aim of this study was to compare the growth characteristics, growing medium properties, and leaf nutrient contents of *S. compacta* seedlings grown for two different growing periods under a single pot type, a uniform growing medium, and a standardized fertilization program. Ultimately, this study aims to provide practical insights into optimizing production duration, which may offer significant advantages for the efficiency and management of commercial nursery operations.

## 2. Results

### 2.1. Changes in Plant Growth Characteristics over Time

**Figure 1:**
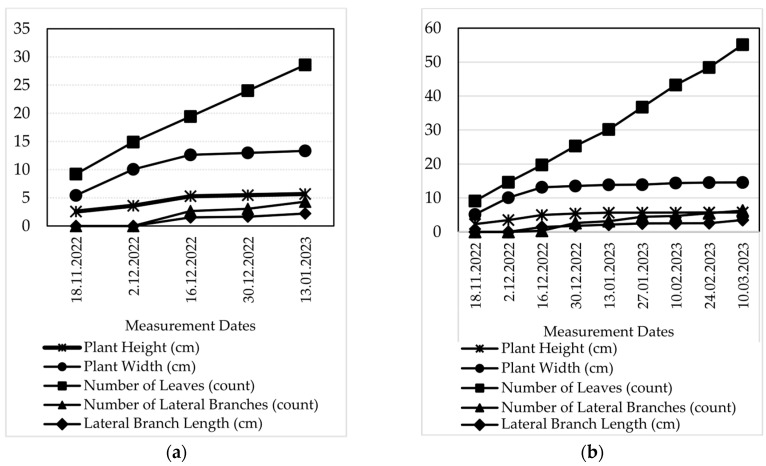
Changes in plant growth characteristics of *Silene compacta* Fisch. over time: (**a**) growth period of 2.5 months; (**b**) growth period of 4.5 months.

As shown in Table 1, five measurements were taken for plant growth characteristics during the 2.5-month growing period, whereas nine measurements were taken during the 4.5-month growing period in *Silene compacta* Fisch. Figure 1 presents the temporal changes in plant growth characteristics for both growing periods.

In both growing periods, plant height, plant width, leaf number, number of lateral branches, and lateral branch length increased markedly during the early phase of growth. In the 2.5-month growing period, plant height and plant width approached a plateau toward the final measurements, while leaf number and lateral branch development continued to increase. A similar pattern was observed in the 4.5-month growing period, although the rate of increase became more limited in the later measurements, particularly for plant height and plant width.

### 2.2. Growth Characteristics, Growing Medium Properties, and Leaf Nutrient Contents According to Different Growing Periods

#### 2.2.1. Growth Characteristics by Growing Period

Table 2 presents the growth characteristics of *Silene compacta* Fisch. at the end of the two growing periods, together with the statistical comparisons (mean ± SD, independent samples *t*-test).

**Figure 2:**
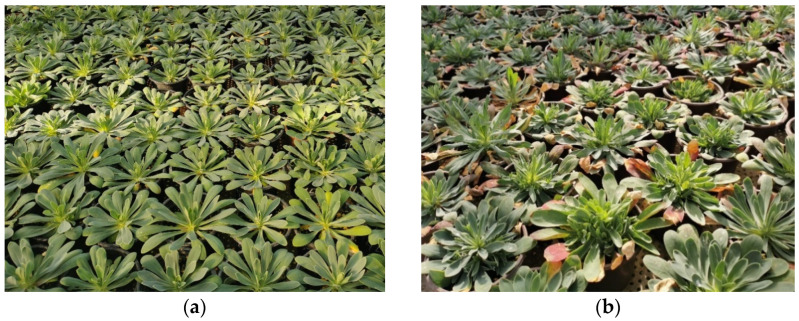
Comparative morphological development of *Silene compacta* Fisch. plants at different growth periods: (**a**) 2.5 months of growth; (**b**) 4.5 months of growth.

Statistically significant differences were detected between the 2.5-month and 4.5-month growing periods for several growth traits. Leaf number (*p* = 0.006), leaf area (*p* = 0.033), root number (*p* = 0.006), fresh root weight (*p* < 0.001), fresh stem weight (*p* = 0.013), dry root weight (*p* < 0.001), dry stem weight (*p* < 0.001), and stem diameter (*p* < 0.001) were significantly higher at 4.5 months than at 2.5 months. By contrast, plant height (*p* = 0.227), plant width (*p* = 0.110), number of lateral branches (*p* = 0.072), lateral branch length (*p* = 0.119), leaf width (*p* = 0.617), leaf length (*p* = 0.159), and root length (*p* = 0.250) did not differ significantly between the two growing periods. The overall visual appearance and morphological differences between the seedlings cultivated under the two distinct periods are explicitly illustrated in Figure 2.

#### 2.2.2. Growing Medium Properties According to Growing Periods

Table 3 presents the growing medium properties of *Silene compacta* Fisch. at the end of the 2.5- and 4.5-month growing periods.

Among the analyzed growing medium properties, pH was significantly higher at 4.5 months than at 2.5 months (*p* = 0.016), whereas total nitrogen content and CEC were significantly lower at 4.5 months (*p* = 0.002 and *p* = 0.002). In addition, available Mg, Fe, Mn, Zn, and Cu concentrations were significantly lower at 4.5 months than at 2.5 months (*p* < 0.05 or *p* < 0.001, depending on the variable). No significant differences were detected between the two growing periods for bulk density, porosity, water-holding capacity, EC, organic matter, available *p*, available K, or available Ca (*p* > 0.05).

#### 2.2.3. Leaf Nutrient Contents According to Growing Periods

Table 4 presents the leaf nutrient contents of *Silene compacta* Fisch. at the end of the two growing periods, together with their statistical comparisons (mean ± SD, *t*-test results).

Significant differences between growing periods were observed for several leaf nutrient elements. Leaf N (*p* < 0.001), P (*p* = 0.020), K (*p* = 0.003), Mn (*p* = 0.019), and Cu (*p* < 0.001) concentrations were significantly higher at 2.5 months than at 4.5 months, whereas Ca concentration was significantly higher at 4.5 months (*p* = 0.016). Leaf Mg (*p* = 0.539), Fe (*p* = 0.770), and Zn (*p* = 0.121) concentrations did not differ significantly between the two growing periods.

## 3. Discussion

Due to the limited number of studies on *Silene compacta* Fisch. in the literature, the results of the present study are discussed in comparison with findings from other plant species to elucidate the underlying physiological mechanisms. The results of this study showed that the growth characteristics of *S. compacta* seedlings differed according to growing period. While several traits—including leaf number, leaf area, root number, stem diameter, and root and shoot biomass—were significantly higher at 4.5 months, no significant differences were detected between the two growing periods for plant height, plant width, number of lateral branches, lateral branch length, leaf length, leaf width, or root length. These findings indicate that extending the growing period from 2.5 to 4.5 months was associated with increases in several biomass-related and structural traits, whereas several basic morphological traits remained statistically similar between the two growing periods. The finding that a shorter production period can still provide marketable plant quality is in line with the study by Carapezza et al. [18] on *Garberia heterophylla*, which aimed to achieve “the highest plant quality in the shortest possible time.” It has been reported that for many woody seedlings grown in approximately 1 L (quart-sized) pots, the typical greenhouse period can range from 8 to 12 weeks depending on the species and substrate [18,26,27]. In this context, the 2.5-month growing period of *S. compacta* appears to provide substantial seedling development within a relatively short production time. This aligns with our primary objective to optimize cultivation duration, demonstrating that a shorter production cycle can effectively meet commercial quality standards while improving operational efficiency. However, for some slow-growing species like *Garberia*, it has been noted that 5–6 months may be required for full root system development [18]. This highlights that interspecific differences directly influence the required cultivation duration.

The growing medium properties also differed between the two growing periods. Statistically significant differences were observed between the periods in terms of total nitrogen, magnesium, iron, manganese, zinc, and copper contents, with these nutrients being higher at 2.5 months than at 4.5 months. On the other hand, water-holding capacity, potassium, and calcium contents were higher at 4.5 months, although these differences were not statistically significant. Moreover, no significant differences were observed between the periods in terms of bulk density, porosity, organic matter, and cation exchange capacity. At the end of the 4.5-month period, pH was higher, whereas total nitrogen and several micronutrients were lower than at 2.5 months. These differences may reflect changes in substrate conditions over the longer cultivation period under repeated irrigation and fertilization. Many studies emphasize that the pH and electrical conductivity (EC) values of the growing medium are critical for nutrient uptake and overall plant development [18,24,28,29,30,31]. For most nursery plants, a pot substrate pH ranging from 5.4 to 6.5 is recommended, representing the range in which plant nutrients are most available in solution [18,32]. In the present study, the increase in pH observed at the longer growing period may have contributed to differences in nutrient availability within the growing medium.

The leaf nutrient contents of *S. compacta* seedlings also differed according to growing period. Statistically significant differences were observed between periods in terms of nitrogen (N), phosphorus (P), potassium (K), manganese (Mn), and copper (Cu) contents, with these nutrients being higher in seedlings grown for 2.5 months compared to 4.5 months. Calcium (Ca) content, however, was higher in the 4.5-month period. No statistically significant differences were observed between periods for magnesium (Mg), iron (Fe), and zinc (Zn) contents. Similar to our findings regarding nutrient dynamics over growth periods, Duan et al. [33] emphasized that evaluating seasonal nutrient fluctuations is critical for determining the optimal nutritional diagnostic and management periods in *Paeonia ostii*, a perennial ornamental species. Similarly, Niklas [19] emphasized that high leaf N and P contents indicate faster growth rates in plants. Likewise, Wang et al. [20] reported that leaf N and P contents significantly affect plant metabolism and growth processes. Furthermore, in the 2.5-month growth period, not only N, P, and K but also other nutrients were found at higher levels compared to the 4.5-month period. Supporting this result, Kumar et al. [34] highlighted that macronutrients interact not only among themselves but also with micronutrients, mutually influencing metabolic processes in the plant. Together, these findings suggest that nutrient accumulation patterns in *S. compacta* varied between the two growing periods.

As is well known, leaf nutrient contents are influenced by numerous factors, including the genetic makeup of the plant material, the physical and chemical properties of the growing medium, light conditions, fertilization, and irrigation regimes [11,35]. In the present study, differences observed between the two growing periods in both substrate and leaf nutrient contents may be related to differences in cultivation duration and the cumulative number of irrigation and fertilization applications. In the short growth period (5 fertilizations + 10 irrigations), although the amount of fertilizer applied was limited, the lower number of irrigations likely reduced nutrient losses from the substrate. In contrast, during the long growth period (9 fertilizations + 18 irrigations), the higher total number of irrigations may have contributed to greater nutrient depletion from the growing medium despite the greater number of fertilizer applications. Warsaw et al. [12] reported that nitrate and phosphate losses are highest during irrigation in container-grown plants. These findings suggest that, particularly under a longer cultivation period, coordinated management of irrigation and fertilization is critical to maintaining nutrient balance. Accordingly, the nutrient differences observed at 2.5 and 4.5 months should be interpreted as responses associated with production period under the applied nursery conditions rather than as direct evidence of isolated nutrient effects. Ultimately, these results confirm that the 2.5-month period is a viable strategy for achieving satisfactory growth with reduced resource inputs, thereby supporting the objective of this study to enhance the economic sustainability of *S. compacta* nursery production.

## 4. Materials and Methods

### 4.1. Planting Material and Experimental Site Conditions

Seedlings of *Silene compacta* Fisch. obtained from seeds were used as the plant material in this study. Seeds were collected from a natural population in Cevizli Village, Akseki District, Antalya, Türkiye. The seeds were sown on 7 October 2022 in trays filled with a peat:perlite mixture (1:1, *v*/*v*) under greenhouse conditions. After the seedlings had developed 4–5 true leaves, uniform seedlings were selected and transplanted on 4 November 2022 into pots with a top diameter of 10.5 cm containing a peat:perlite growing medium (2:1, *v*/*v*). No additional materials or lime were added to the growing medium.

**Figure 3:**
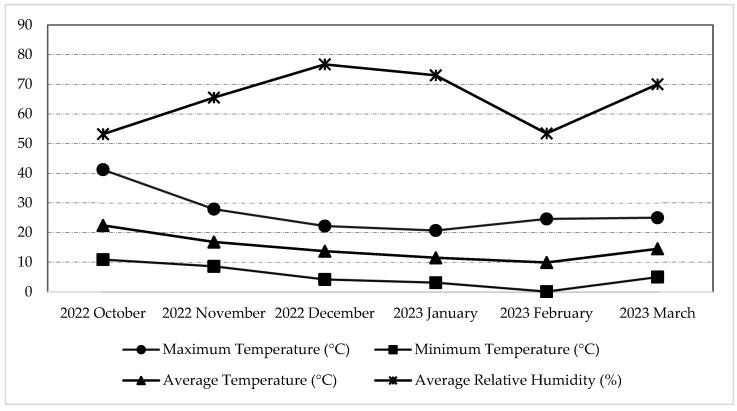
Greenhouse climate parameters for *Silene compacta* Fisch.

The experiment was conducted in a greenhouse under the same production conditions for all plants. Greenhouse climate data during the experimental period are presented in Table 5 and Figure 3. During the study period, monthly maximum temperature ranged from 20.7 to 41.2 °C, monthly minimum temperature ranged from 0.1 to 10.9 °C, monthly average temperature ranged from 9.9 to 22.4 °C, and monthly average relative humidity ranged from 53.2% to 76.7%.

### 4.2. Experimental Design and Growing Procedures

The experiment was established as a single-factor study to compare two growing periods: 2.5 months and 4.5 months. A total of 180 seedlings were used. At the time of transplanting, uniform seedlings derived from the same sowing batch were randomly assigned to two growing-period groups. Each growing-period treatment consisted of 90 plants arranged in three replicates, with 30 plants per replicate.

The seedlings assigned to the 2.5-month and 4.5-month growing periods were different plants, but all originated from the same sowing date, were transplanted on the same date, and were grown simultaneously under the same greenhouse conditions. At the beginning of the experiment, plants were randomly allocated to the two growing-period groups and to the three replicates within each group. All pots were placed on the same production bench. The two growing-period groups were arranged separately on the same bench, and the replicates within each group were positioned side by side. Pot positions were not changed during the experimental period.

All plants were grown in pots of the same size and in the same growing medium, and all received the same fertilization program throughout their assigned growing period. Fertilization was applied every two weeks as 10 mL per pot of a nutrient solution containing N, P, and K at 100, 50, and 150 ppm, respectively. The fertilizer solution was prepared using monoammonium phosphate (10-52-10), potassium nitrate (13-0-46), and urea (46-0-0). Plants in the 2.5-month treatment received a total of five fertilizer applications, and plants in the 4.5-month treatment received a total of nine fertilizer applications, applied at two-week intervals.

Irrigation was applied manually once per week to all plants, with 100 mL water supplied per pot at each irrigation event. A total of 10 irrigations were applied during the 2.5-month growing period and 18 irrigations during the 4.5-month growing period. Slight drainage from the bottom of the pots was observed after irrigation.

Final measurements were made at the end of each assigned growing period. The 2.5-month group was evaluated on 13 January 2023, and the 4.5-month group was evaluated on 10 March 2023.

### 4.3. Growth Measurements and Sample Collection

During both growing periods, plant height, plant width, leaf number, number of lateral branches, and lateral branch length were measured at two-week intervals to monitor temporal changes in growth. These data were used to generate growth curves for descriptive presentation.

At the end of each growing period, the following morphological traits were measured for the plants in both groups: plant height (cm), plant width (cm), leaf number, number of lateral branches, lateral branch length (cm), stem diameter (mm), leaf width (cm), leaf length (cm), leaf area (cm^2^), root number, root length (cm), fresh root weight (g), fresh stem weight (g), dry root weight (g), and dry stem weight (g).

Plant height was measured from the surface of the growing medium to the highest point of the plant using a ruler. Plant width was measured as the widest part of the plant canopy. Leaf number was determined by counting all leaves on each plant. The number of lateral branches longer than 0.5 cm was counted for each plant, and lateral branch length was measured from the longest lateral branches using a ruler. Stem diameter was measured 0.5 cm above the growing medium surface using a digital caliper.

For leaf measurements, 10 leaves per plant were sampled. Leaf width was measured at the widest point, leaf length at the longest point, and leaf area was determined using a leaf area meter. Root number included all roots longer than 0.5 cm. Root length was measured using a ruler. Fresh root weight and fresh stem weight were measured separately using a balance with 0.01 g precision. For dry weight determination, roots and stems were dried separately at 70 °C for 5 days and then weighed.

At the end of each growing period, leaf samples were collected from all individual plants within each replicate for nutrient analysis, and these samples were thoroughly composite/pooled on a replicate basis to ensure a representative homogeneous mixture. In addition, growing medium samples were collected from the pots of all plants within each replicate for the analysis of physical and chemical properties, and were similarly composite for each replicate group. Laboratory analyses for leaf nutrient concentrations and growing medium properties were conducted strictly on these composite replicate frameworks, yielding three independent true replicate values for each growing period duration. The pH and electrical conductivity (EC) of the growing medium were determined in a 1:10 soil/water suspension. Water-holding capacity was assessed using a modified TS-10041 method. Organic matter content was determined through dry combustion (loss on ignition) at 550 °C. Total nitrogen (N) in both leaf tissues and growing medium was analyzed using the Kjeldahl method. Available macro- and micronutrients (P, K, Ca, Mg, Fe, Mn, Zn, Cu) in the growing medium were extracted via water-extraction and measured using Inductively Coupled Plasma (ICP) spectroscopy (PerkinElmer Optima 2100 DV, PerkinElmer, Waltham, MA, USA)Similarly, leaf macro- and micronutrient concentrations (N, P, K, Ca, Mg, Fe, Mn, Zn, Cu) were determined via ICP spectroscopy following wet digestion. Cation exchange capacity (CEC) was determined using sodium saturation followed by ICP analysis. Finally, bulk density and porosity were evaluated according to the modified ASTM 2006 [36] and ASTM D 2980 (2010) [37] standards, respectively.

### 4.4. Statistical Analysis

Each growing period consisted of three replicates, with 30 plants per replicate. For plant growth traits, measurements obtained from individual plants within each replicate were averaged, and replicate means were used as the experimental units for statistical analysis. Accordingly, comparisons between the 2.5-month and 4.5-month growing periods were based on three replicate means per treatment.

Differences between the two growing periods for plant growth traits, leaf nutrient contents, and growing medium properties were evaluated using independent samples *t*-tests, because the 2.5-month and 4.5-month treatments consisted of separate plant groups established at the beginning of the experiment. Statistical significance was assessed at the *p* ≤ 0.05 level. All statistical analyses were performed using IBM SPSS Statistics (Version 20; IBM Corp., Armonk, NY, USA).

Measurements taken at two-week intervals during the growing period were used to describe temporal changes in selected growth traits and were presented descriptively as graphs.

## 5. Conclusions

Under the standardized nursery conditions tested in this study, the findings indicate that a 2.5-month growing period provides sufficient seedling development within a shorter production time for *Silene compacta* Fisch. Although leaf number, leaf area, root number, stem diameter, and root and shoot biomass were higher at 4.5 months, no significant differences were detected between the two growing periods in plant height, plant width, number and length of lateral branches, leaf length, leaf width, or root length. These results suggest that the 2.5-month period may represent a practical option for producing seedlings with a compact appearance, adequate basic morphological development, and a shorter production cycle.

In addition, the 2.5-month period appears to be more advantageous in terms of production cost, as it requires fewer irrigation and fertilization applications. The shorter production time may also allow an earlier saleable appearance, a more efficient use of nursery space, and a shorter turnover period in nursery production. The higher concentrations of some leaf nutrient elements at 2.5 months further suggest that this period may be noteworthy in terms of nutrient use and seedling quality. Consequently, by optimizing the cultivation duration to 2.5 months, producers can achieve high-quality seedlings while significantly improving resource use efficiency and economic viability. Overall, the 2.5-month growing period stands out as a shorter, more compact, and economically favorable production period for *S. compacta* seedlings under the nursery conditions tested, and the present study provides practical baseline information for its nursery production, thereby addressing the critical need for efficient and cost-effective cultivation protocols in the ornamental plant industry.

## Figures and Tables

**Table 1:** Temporal growth characteristics of *Silene compacta* Fisch. at 2.5- and 4.5-month growth periods (3 replicates per treatment).

Plant Growth Characteristics	Growth Period	Measurement Dates								
		18.11.22	2.12.22	16.12.22	30.12.22	13.01.23	27.01.23	10.02.23	24.02.23	10.03.23
Plant height (cm)	2.5 month	2.56	3.59	5.28	5.47	5.67				
	4.5 month	2.30	3.48	4.97	5.41	5.66	5.68	5.70	5.72	5.73
Plant width (cm)	2.5 month	5.43	10.05	12.62	12.97	13.33				
	4.5 month	5.14	10.08	13.14	13.51	13.85	13.91	14.37	14.52	14.56
Number of leaves (count)	2.5 month	9.20	14.89	19.42	24.01	28.58				
	4.5 month	9.08	14.62	19.71	25.29	30.14	36.72	43.25	48.41	55.11
Number of lateral branches (count)	2.5 month	0	0	2.65	3.04	4.31				
	4.5 month	0	0	0.32	2.62	3.16	4.46	4.70	5.49	6.27
Lateral branch length (cm)	2.5 month	0	0	1.52	1.65	2.21				
	4.5 month	0	0	1.44	1.79	2.12	2.48	2.52	2.57	3.50

**Table 2:** Growth characteristics of *Silene compacta* Fisch. at different growth periods and their statistical differences (3 replicates per treatment, mean ± SD. *t*-test results).

Plant Growth Characteristics	Growth Period		*t*-Value	*p*-Value
	2.5 Months	4.5 Months		
Plant height (cm)	5.670 ± 0.35	5.73 ± 0.26	−1.427	0.227
Plant width (cm)	13.33 ± 0.77	14.56 ± 0.60	−2.048	0.110
Number of leaves (count)	28.58 ± 0.50	55.11 ± 9.02	−5.389	0.006
Leaf area (cm)	6.93 ± 0.35	8.08 ± 0.52	−3.189	0.033
Number of lateral branches (count)	4.31 ± 1.15	6.27 ± 1.59	−2.434	0.072
Lateral branch length (cm)	2.21 ± 0.34	3.50 ± 0.96	−1.975	0.119
Stem diameter (mm)	5.22 ± 0.19	7.55 ± 0.19	−14.978	0.000
Leaf width (cm)	1.91 ± 0.16	1.98 ± 0.14	−0.542	0.617
Leaf length (cm)	7.48 ± 0.35	8.11 ± 0.53	−1.726	0.159
Number of roots (count)	16.20 ± 0.35	18.55 ± 0.69	−5.264	0.006
Root length (cm)	18.60 ± 0.66	20.28 ± 2.05	−1.345	0.250
Fresh root weight (g)	1.29 ± 0.11	5.01 ± 0.32	−18.996	0.000
Fresh stem weight (g)	8.87 ± 0.56	12.71 ± 1.43	−4.307	0.013
Dry root weight (g)	0.24 ± 0.03	0.94 ± 0.08	−14.560	0.000
Dry stem weight (g)	1.22 ± 0.03	2.79 ± 0.25	−10.806	0.000

**Table 3:** Growing medium contents of *Silene compacta* Fisch. at different growth periods and their statistical differences (3 replicates per treatment, mean ± SD, *t*-test results).

Growing Medium Properties	Growth Period		*t*-Value	*p*-Value
	2.5 Months	4.5 Months		
Bulk density (g/cm^3^)	0.14 ± 0.01	0.15 ±0.00	−2.000	0.116
Porosity (%)	61.44 ± 2.13	59.26 ± 2.54	1.430	0.226
Water-holding capacity (%)	637.73 ± 60.59	1272.08 ± 535.20	−2.040	0.111
pH	7.23 ± 0.12	7.5 ± 0.00	−4.000	0.016
EC (µS/cm)	139.33 ± 11.59	116.73 ± 12.95	2.253	0.087
Organic matter (%)	56.53 ± 16.22	67.17 ± 8.37	−1.009	0.370
CEC (me/100 g)	21.33 ± 1.89	13.73 ± 0.29	6.940	0.002
Total Nitrogen (N) (%)	0.38 ± 0.04	0.28 ± 0.00	7.460	0.002
Available Phosphorus (P) (ppm)	5.6 ± 4.67	2.13 ± 2.56	1.256	0.277
Available Potassium (K) (ppm)	26.85 ± 29.43	46 ± 40.57	−0.662	0.544
Available Calcium (Ca) (ppm)	89.83 ± 8.92	114.77 ± 18.30	−2.121	0.101
Available Magnesium (Mg) (ppm)	31.33 ± 2.78	15.37 ± 2.08	7.961	0.001
Available Iron (Fe) (ppm)	1.68 ± 0.13	0.05 ± 0.04	21.039	0.000
Available Manganese (Mn) (ppm)	0.67 ± 0.01	0.01 ± 0.00	197.000	0.000
Available Zinc (Zn) (ppm)	0.65 ± 0.02	0.02 ± 0.00	72.191	0.000
Available Copper (Cu) (ppm)	0.68 ± 0.01	0.03 ± 0.00	202.000	0.000

**Table 4:** Leaf nutrient contents of *Silene compacta* Fisch. at different growth periods and their statistical differences (3 replicates per treatment, mean ± SD, *t*-test results).

Leaf Nutrient Elements	Growth Period		*t*-Value	*p*-Value
	2.5 Months	4.5 Months		
Nitrogen (N) (%)	2.74 ± 0.15	1.54 ± 0.09	12.020	0.000
Phosphorus (P) (%)	0.27 ± 0.06	0.11 ± 0.03	3.731	0.020
Potassium (K) (%)	2.01 ± 0.21	1.01 ± 0.18	6.244	0.003
Calcium (Ca) (%)	0.85 ± 0.13	1.37 ± 0.18	−4.018	0.016
Magnesium (Mg) (%)	0.93 ± 0.11	0.98 ± 0.06	−0.670	0.539
Iron (Fe) (ppm)	56.37 ± 15.68	53.33 ± 6.05	0.313	0.770
Manganese (Mn) (ppm)	47.20 ± 5.82	32.30 ± 3.51	3.797	0.019
Zinc (Zn) (ppm)	47.37 ± 8.46	57.60 ± 3.12	−1.966	0.121
Copper (Cu) (ppm)	10.53 ± 0.80	1.23 ± 0.70	15.109	0.000

**Table 5:** Climate parameters under greenhouse conditions of *Silene compacta* Fisch.

Climate Parameters	The Year 2022			The Year 2023		
	October	November	December	January	February	March
Maximum Temperature (°C)	41.20	27.90	22.20	20.70	24.60	25.00
Minimum Temperature (°C)	10.90	8.60	4.20	3.10	0.10	5.00
Average Temperature (°C)	22.40	16.80	13.70	11.50	9.90	14.50
Average Relative Humidity (%)	53.20	65.50	76.70	73.00	53.40	70.00

## Data Availability

The data generated and analyzed during the current study are available from the corresponding author upon reasonable request.
